# Novel genome-editing-based approaches to treat motor neuron diseases: Promises and challenges

**DOI:** 10.1016/j.ymthe.2021.04.003

**Published:** 2021-04-03

**Authors:** Annarita Miccio, Panagiotis Antoniou, Sorana Ciura, Edor Kabashi

**Affiliations:** 1 Laboratory of Chromatin and Gene Regulation during Development, Imagine Institute, Université de Paris, INSERM UMR 1163, 75015, Paris, France; 2 Laboratory of Translational Research for Neurological Disorders, Imagine Institute, Université de Paris, INSERM UMR 1163, 75015, Paris, France

**Keywords:** motor neuron diseases, SMA, ALS, genome editing, CRISPR, base editing

## Abstract

Motor neuron diseases are untreatable with common pharmacological approaches. Spinal muscular atrophy (SMA) is caused by *SMN1* gene mutations leading to lowered SMN expression. Symptoms are alleviated in infants with a higher copy number of the *SMN2* gene, which, however, displays a splicing defect resulting in low SMN levels. Amyotrophic lateral sclerosis (ALS) is caused by a number of mutations, with *C9orf72* repeat expansions the most common genetic cause and *SOD1* gain-of-function mutations the first genetic cause identified for this disease. Genetic therapies based on oligonucleotides that enhance *SMN2* splicing and SMN production or lower *SOD1* expression have shown promise in initial clinical trials for individuals with SMA and ALS harboring SOD1 mutations, respectively. Gene addition/silencing approaches using adeno-associated viruses (AAVs) are also currently under clinical investigation in trials for SMA and ALS. Here we provide a brief overview of these efforts and their advantages and challenges. We also review genome editing approaches aimed at correcting the disease-causing mutations or modulating the expression of genetic modifiers, e.g., by repairing *SOD1* mutations or the *SMN2* splicing defect or deleting *C9orf72* expanded repeats. These studies have shown promising results to approach therapeutic trials that should significantly lower the progression of these deadly disorders.

## Graphical abstract

**Figure:**
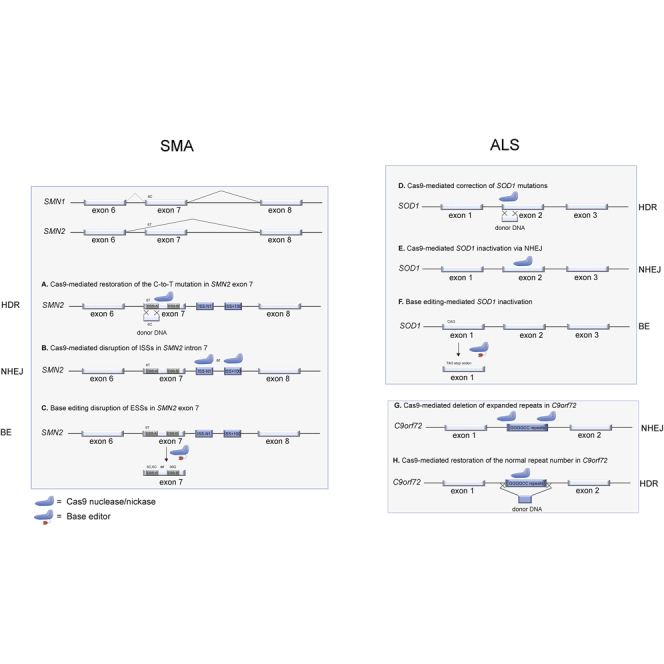

## Teaser

Miccio et al. provide a brief overview of the current genetic strategies for two major motor neuron diseases: spinal muscular atrophy and amyotrophic lateral sclerosis. The authors also discuss novel genome editing-based approaches and their challenges and advantages.

## Introduction

Motor neuron diseases lead to motor neuron degeneration and muscle wasting and have devastating consequences for affected individuals. Here we introduce the main genetic features of the two most prevalent motor neuron diseases: spinal muscular atrophy (SMA) and amyotrophic lateral sclerosis (ALS).

SMA is the most common disease leading to child mortality with an incidence of 1 in 10,000 births.^1^ This autosomal recessive disease is caused by homozygous loss-of-function mutations in the *SMN1* gene (biallelic deletions in exon 7 and/or 8 in 95% of cases).^2^ Importantly, a paralogous gene, *SMN2*, encodes the same protein, SMN. However, the majority of the *SMN2*-derived SMN protein is nonfunctional as a result of aberrant splicing. The main difference between *SMN1* and *SMN2* is a C-to-T transition in a splice modulator located in exon 7; this mutation inhibits exon 7 inclusion in the mRNA, causing production of a truncated protein called SMNΔ7.^3^^,^^4^ In fact, loss-of function *SMN1* mutations can occur via *SMN1-to-SMN2* gene conversion. Nevertheless, *SMN2* still produces 10% of full-length transcripts. Indeed, the levels of *SMN2* transcripts correlate directly with the clinical stage of SMA, and the severity of symptoms is alleviated in infants with a higher copy number of the *SMN2* gene.^5^^,^^6^

ALS is the most prevalent adult-onset motor neuron disease with an estimated incidence of 1.9 per 100,000 births.^7^. There is an urgent need for more efficient treatments for this devastating disease; riluzole, a US Food and Drug Administration (FDA)-approved drug for ALS, offers only a modest survival benefit.^8^ The first described mutations in ALS affect the *SOD1* gene, causing formation of a neurotoxic mutant protein.^9^ Importantly, a mouse model overexpressing mutant SOD1 has been shown to recapitulate a number of ALS features, including paralysis and muscle wasting.^10^ In 2011, the most prevalent genetic cause of ALS was identified as an expansion of hexanucleotide repeats in the *C9orf72* gene.^11^^,^^12^ Toxic gain of function of the expanded repeats has been considered as a pathogenic mechanism because RNA foci and dipeptide repeats resulting from transcription and translation of hexanucleotide repeats are found in pathological samples from individuals with ALS.^13^ On the other hand, significantly reduced levels of *C9orf72* transcript and protein as well as promoter methylation have been described in tissues and cells obtained from individuals with ALS, indicating that C9orf72 loss of function could lead to neurodegeneration.^14^^,^^15^

Both of these major neurological diseases lead to a severe handicap and cause early mortality in the majority of affected people. These disorders are currently untreatable with common pharmacological approaches. On the other hand, genetic therapy has been quite promising in early clinical trials, particularly for SMA.^16^^,^^17^ Here we provide a brief overview of these initial therapeutic strategies and cover the pre-clinical studies exploiting genome editing approaches and their challenges and advantages.

## Conventional genetic therapy approaches to treat SMA and ALS

Modifying *SMN2* splicing to produce a functional SMN protein is a universal therapy for individuals harboring mutations in the *SMN1* gene. Oligonucleotide drugs (developed mainly by Ionis and Biogen) promote increased production of full-length SMN protein by targeting the intronic splicing silencer N1 (ISS-N1) of *SMN2*, inhibiting binding of splicing regulators^18^, ^19^, ^20^ and favoring inclusion of exon 7 in *SMN2*. Oligonucleotides that modify *SMN2* RNA splicing (Spinraza, nusinersen) were clinically approved following major clinical trials undertaken in a number of European and North American centers, with a better clinical outcome upon early intervention.^16^^,^^17^ Even though this approach was successful in a number of cases, this oligonucleotide-based therapy has major side effects, is costly, and needs to be replenished, and a number of people do not benefit from this treatment. Gene addition by adeno-associated virus 9 (AAV9) vectors is currently in a trial for SMA by Novartis (Zolgensma trial), with promising preliminary results,^21^^,^^22^ but the long-term efficacy of this treatment has not yet been determined.

Current genetic therapies for individuals with ALS carrying *SOD1* mutations include administration of antisense oligonucleotides targeting *SOD1*, with a phase 1/2 trial reported by Miller et al.^23^ *SOD1* gene silencing strategies using AAVrh10 have also been reported.^24^^,^^25^ Similar genetic therapies featuring oligonucleotides or AAV-mediated gene silencing have been undertaken to target the RNA repeats of the C9orf72 gene (reviewed in Cappella et al.^25^). However, these strategies remain temporary and need replenishing (as in the case of oligonucleotides) or are still undergoing early clinical trials (for gene delivery via AAVs).

## Genome editing approaches

Given the abovementioned limitations of the current oligonucleotide-based therapies and gene replacement/silencing approaches, there is a strong rationale for developing innovative and definitive gene therapy strategies for SMA and ALS and related neurological disorders.

### Nuclease-based genome editing approaches

Genome editing has been exploited recently to develop therapeutic approaches for genetic and non-genetic diseases. These tools include zinc-finger nucleases (ZFNs), transcription activator-like effector nucleases (TALENs), and the clustered regularly interspaced short palindromic repeats (CRISPR)-associated nuclease Cas9 and enable precise genome editing by inducing DNA double-strand breaks (DSBs) at selected genomic loci. In particular, engineering of the nuclease Cas9-mediated bacterial adaptive immunity response against viruses^26^ provides an easy way of generating sequence-specific DSBs based on a single guide RNA (gRNA) that anneals to the target genomic sequence.^27^, ^28^, ^29^ The DNA sequence (protospacer) targeted by the commonly used SpCas9 nuclease (*Streptococcus pyogenes*) has to be followed immediately by a 3-bp DNA sequence (NGG, called the protospacer-adjacent motif [PAM]) to allow Cas9 binding and cleavage. Other bacterial enzymes requiring different PAMs, such as *Staphylococcus aureus* Cas9 (SaCas9) and *Prevotella* and *Francisella 1* Cpf1, are commonly used to expand the targeting scope of the CRISPR system. The DSBs are then repaired by the cellular repair machinery via homology-directed repair (HDR) or non-homologous end-joining (NHEJ). HDR is a high-fidelity mechanism where homologous DNA serves as a repair template; therefore, by delivering an exogenous donor DNA (dDNA; provided as a single-stranded oligonucleotide [ssODN] or delivered by AAV) flanked by sequences that are homologous to the nuclease target site, it is possible to achieve dDNA site-specific integration and potentially correct disease-causing mutations. However, HDR-based approaches are inefficient in non-dividing cells. Furthermore, failed HDR-mediated gene correction can lead to gene disruption by NHEJ, a more active DNA repair pathway in quiescent cells, generating a knockout phenotype instead of correcting the mutation. CRISPR/Cas9-mediated NHEJ is an error-prone pathway and has mainly been exploited to obtain permanent gene inactivation and disrupt *cis* elements regulating gene expression via generation of insertions or deletions (indels). NHEJ occurs in every phase of the cell cycle; thus, it can be exploited easily to genetically modify a large number of cell types. However, Cas9 nuclease induces DSBs that can lead to apoptosis and large genomic rearrangements (e.g., deletions, inversions, and translocations).^30^ Therefore, use of Cas9 nuclease-based technologies raises safety concerns for clinical applications, including for motor neuron diseases, as discussed in the following section.

### Base editing (BE) and prime editing (PE) approaches

It has been reported recently that CRISPR system-based cytosine and adenine base editors (CBEs and ABEs, respectively) can make pinpoint changes in DNA with little or no DSB generation.^31^, ^32^, ^33^ CBEs convert C-G to T-A, whereas ABEs convert A-T to G-C.^34^ Novel base editors are even able to make C-G-to-G-C transversions.^35^ Base editing approaches allow precise DNA repair in the absence of DSBs, eliminating the likelihood of indels of large chunks of DNA at DSBs, DNA translocations, and DSB-induced toxicity. Furthermore, in some cases, BE has been shown to be more efficient than Cas9 nuclease-mediated HDR and to have less off-target activity than Cas9 nuclease.^31^^,^^32^ Importantly, BE occurs in quiescent cells; thus, it can be exploited to genetically modify non-dividing cells, such as post-mitotic neurons,^36^ and results in homogeneous, predictable base changes compared with the heterogeneous and unpredictable mutagenesis induced by NHEJ. CBEs and ABEs can be used to correct disease-causing mutations and inactivate genes; e.g., by generating nonsense mutations.

Finally, prime editors have been described recently as “search-and-replace” genome editors.^37^ Prime editors “rewrite” genomes by inducing all 12 possible base conversions, indels, or a combination of these changes without inducing DSBs. This novel strategy is based on two main components: a disabled Cas9 nuclease fused to a reverse transcriptase (RT) and a PE gRNA (pegRNA). Different from the BE system, another genome editing system enabling specific pinpoint mutations, prime editors can theoretically induce all possible base conversions.

## Genome editing for motor neuron diseases

The genome editing systems described above are innovative genetic therapy platforms that can be exploited to precisely repair genetic mutations causing motor neuron disorders. In the next section, we review recent advances of these genome editing systems and the recent pre-clinical evidence in SMA and ALS that encourages further clinical work. Furthermore, we review the challenges and perspectives faced to translate these findings into therapeutic advances for these neurological diseases.

### Genome editing strategies for SMA

**Figure 1:**
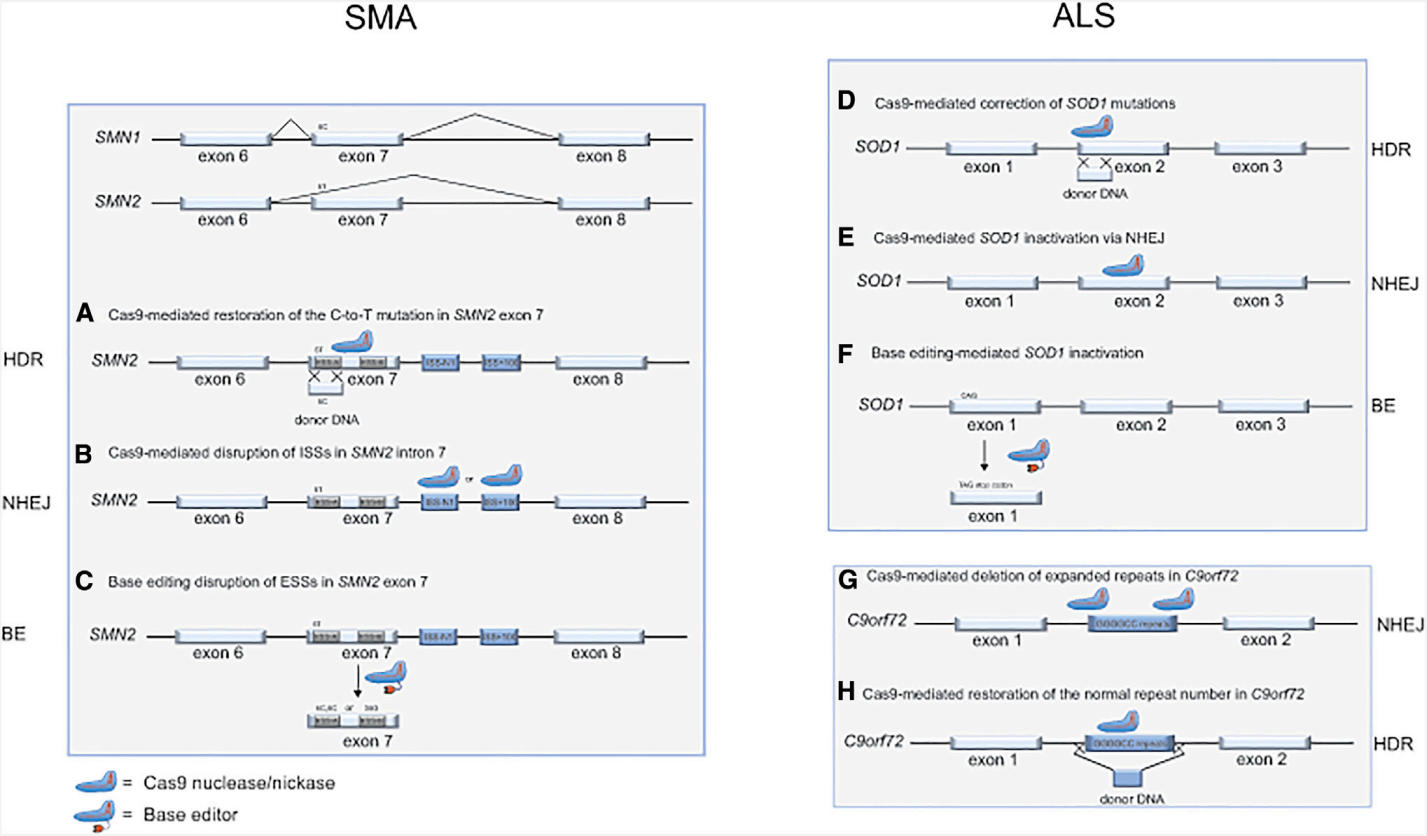
Genome editing approaches for SMA and ALS Left panel: schematic of *SMN1* and *SMN2* gene fragments spanning from exon 6 to exon 8. A single-nucleotide difference (C-to-T transition) in *SMN2* causes exon 7 skipping and production of a defective protein. Genome editing approaches for SMA include the following. (A) Cas9 nuclease-mediated restoration of the C-to-T mutation in position 6 of *SMN2* exon 7 via HDR; the donor template harbors the C in position 6 of exon 7. (B) Cas9 nuclease-mediated disruption of ISSs in *SMN2* intron 7 via NHEJ; 5C and 6C or 36G mutations generated by base editors restore the correct *SMN2* splicing. (C) Base editing (BE) disruption of ESSs in *SMN2* exon 7. Right panel: genome editing approaches for individuals with ALS harboring *SOD1* mutations include the following. (D) Cas9 nuclease-mediated correction of the disease-causing mutations via HDR; the donor template harbors the wild-type *SOD1* sequence. (E) Cas9 nuclease-mediated *SOD1* inactivation via NHEJ. (F) *SOD1* inactivation using BE. BE allows conversion of a CAG triplet in a TAG stop codon. Genome editing approaches for individuals with ALS harboring an expansion of GGGGCC repeats in *C9orf72* intron 1 include (G and H) Cas9 nuclease-mediated deletion of the expanded repeats using two gRNAs cleaving the 5′ and 3′ ends of the target region (G) and Cas9 nuclease-mediated correction of *C9orf72* using a donor template containing a normal number of repeats (H). The blue shape indicates Cas9 nuclease or Cas9 nickase. The base editor is composed of a Cas9 nickase fused to a deaminase enzyme (red shape). HDR, homology-directed repair, NHEJ, non-homologous end-joining; ESS-A, exonic splicing silencer A; ESS-B exonic splicing silencer B; ISS-N1, intronic splicing silencer N1; ISS +100, intronic splicing silencer +100.

Recently, genome editing approaches aimed at correcting the splicing of *SMN2* have been explored. Indeed, a genome editing strategy has been developed by several teams to correct the C-to-T mutation in position 6 of *SMN2* exon 7 (Figure 1A).^38^, ^39^, ^40^ Interestingly, transfection of a ssODN donor containing a functional exon 7 without any nuclease restored *SMN2* splicing in a SMA skin fibroblast cell line and in induced pluripotent stem cells (iPSCs) from individuals with SMA.^38^^,^^39^ Motor neurons generated from corrected iPSCs show relevant SMN expression and amelioration of the pathological phenotype *in vitro*. Furthermore, transplantation of corrected motor neurons in SMA transgenic mice improves the disease phenotype.^39^ To increase gene correction efficiency, Zhou and colleagues^40^ delivered the CRISPR/Cpf1 nuclease system (using a gRNA targeting exon 7) combined with a ssODN donor into SMA iPSCs. Restoration of SMN expression in iPSC-derived motor neurons improved the SMA cellular phenotype.^40^ However, HDR-based approaches, even in the presence of a designer nuclease, are usually inefficient (≤1%) and complex because they require delivery of a donor template. Furthermore, failed HDR-mediated gene correction can lead to gene disruption by NHEJ,^40^ potentially worsening the SMN phenotype instead of correcting the *SMN2* mutation.

Interestingly, a CRISPR/Cas9 nuclease approach has been developed to disrupt ISS regions (ISS-N1 and ISS +100) of *SMN2* via NHEJ (Figure 1B). This strategy proved to be efficient in iPSCs from individuals with SMA and in a SMA mouse model after germline correction.^41^ Restoration of correct *SMN2* splicing and SMN production ameliorated the phenotype of iPSC-derived motor neurons and the lifespan and pathological phenotype of SMA mice. This type of approach is likely more efficient than HDR-based strategies and does not need delivery of a donor template. Furthermore, because it targets intronic regions, it avoids the risk of gene inactivation by NHEJ. However, different indels are associated with a different degree of correction of the pathological phenotype, suggesting that use of genetic tools generating predictable mutations are highly desirable to achieve a therapeutic benefit.^41^

To avoid unpredictable mutations generated by designer nucleases, a BE strategy has been explored recently.^42^ In particular, Lin and colleagues^42^ used ABEs to target exonic splicing silencer A (ESS-A; containing the C-to-T transition in position 6 of wild-type *SMN2*) or ESS-B in exon 7 of *SMN2* in SMA iPSCs (Figure 1C). Targeting of ESS-A led to several mutations, including reversion of the C-to-T transition in position 6. However, the major event was a T-to-C mutation in position 5, which led to a Phe > Ser amino acid substitution. Interestingly, this missense transition not only restored splicing but was also effective in ameliorating the cellular phenotype of iPSC-derived motor neurons and prolonging the lifespan and improving the phenotype of germline-edited SMA mice.^42^ Furthermore, targeting of ESS-B led to generation of the synonymous A36G mutation, which ameliorated the cellular phenotype of SMA iPSC-derived motor neurons.^42^

### Genome editing strategies for ALS

HDR-based genome editing approaches have been used to correct a variety of *SOD1* mutations in iPSCs from individuals with ALS (reviewed in Yun and Ha^43^), demonstrating rescue of the ALS cellular phenotype in motor neurons derived from gene-corrected iPSCs (Figure 1D). Similar approaches have been exploited in iPSCs from people with mutations in *FUS* and *TARDBP.*^43^ As expected, efficiency was low (usually less than 1%), but use of selection markers in the donor template combined with screening of a high number of clones allowed selection of gene-corrected iPSCs.

Two groups have used a CRISPR/Cas9 nuclease-based approach to knock out, by NHEJ, the *SOD1* gene harboring the gain-of-function G93A mutation in a transgenic mouse model of ALS (G93A-SOD1 mice) (Figure 1E).^44^^,^^45^ Treatment of neonatal mice with AAV9 expressing SaCas9 (which is smaller than SpCas9 and can fit in the AAV genome) and a gRNA targeting the *SOD1* coding region led only to some therapeutic benefits (e.g., increased survival and reduced muscular atrophy), likely because of inefficient genome editing *in vivo*. Interestingly, Dr. Gaj’s group^46^ proposed using CBEs to generate nonsense mutations in *SOD1* in G93A-SOD1 mice (Figure 1F). The challenge here was to deliver the large CBE enzyme *in vivo* because it does not fit in the AAV vector. Therefore, Gaj and colleagues^46^ developed a split-intein system based on use of two AAV9 vectors bearing the two moieties of a SpCas9-based CBE fused to intein fragments that are reassembled *in vivo* via *trans*-splicing. Injection of AAV9s expressing the CBE and a gRNA targeting *SOD1* in adult mice led to persistent CBE expression and delayed disease manifestation despite the low BE frequency, which was likely due to poor transduction by the dual vectors and the low efficiency of the split-intein system.^46^^,^^47^ Notably, Gaj and colleagues^46^ reported detectable editing of non-target bases (“bystander editing”), which could lead to generation of aberrant mutant proteins.^46^ Finally, CRISPR-based approaches downregulating *SOD1* do not distinguish between mutant and wild-type genes,^44^^,^^46^ but they target regions that are not reported to contain ALS-causing mutations to provide a universal therapy for individuals with ALS harboring mutations in *SOD1.* However, for the same reason, these strategies could be associated with potentially dangerous *SOD1* downregulation, which needs to be taken into account because a homozygous SOD1-truncating mutation abolishing SOD1 activity has been reported to lead to progressive loss of motor activity in an infant.^48^ On the other hand, individuals with ALS treated with SOD1-directed antisense oligonucleotides did not show toxic effects because of *SOD1* downregulation.^24^ Similarly, strategies targeting *FUS* or *TARDBP* using antisense oligonucleotides or genome editing tools should be evaluated carefully to avoid FUS or TDP-43 loss of function, which has been shown to have deleterious effects *in vivo* and *in vitro*.^49^^,^^50^

CRISPR-based approaches have been developed to delete the expanded repeats in the *C9orf72* gene (Figure 1G). Pribadi and colleagues,^51^ Selvaraj et al.,^52^ and Lopez-Gonzalez et al.^53^ used a pair of gRNAs flanking the repeats to excise the region by NHEJ in iPSCs from individuals with ALS. The deletion frequency ranged between 0.6%–11% in unselected bulk iPSC populations.^51^^,^^52^ Edited iPSC clones were isolated and harbored deletion of most of the repeats in the mutant allele, which, in some cases, was larger than expected. Furthermore, the wild-type allele often also showed small deletions. Surprisingly, despite these undesired events, *C9orf72* expression appeared not to be negatively affected. On the other hand, removal of the expanded repeats did diminish formation of RNA foci and dipeptide repeat proteins in these iPSC-derived neurons. Similarly, the authors reported correction of *C9orf72* promoter methylation as well as reduced excitotoxicity, which is typical for ALS motor neurons.^51^, ^52^, ^53^ Recently, two groups used an HDR-based approach to introduce a donor template containing a normal number of repeats in *C9orf72* in ALS iPSCs (Figure 1H).^54^^,^^55^ In particular, they used the Cas9 nickase enzyme, which requires 2 gRNAs to cleave the target site, reducing potential off-targets effects.^56^ The advantage of this HDR-based strategy is the predictable outcome, which avoids generation of deletions of variable extent; however, it does not avoid targeting of the wild-type allele and is not efficient in bulk populations of ALS iPSCs, often requiring introduction in the target site of a selectable marker.^54^^,^^55^ Selected edited ALS iPSC lines showed normal *C9orf72* mRNA expression and promoter methylation.^54^ Motor neurons derived from edited ALS iPSCs showed a reduction of RNA foci and aggregations of dipeptide repeat proteins and improved cell survival and response to cellular stress.^54^ Finally, a CRISPR/Cas9-based strategy to delete *C9orf72* expanded repeats *in vivo* has been reported recently.^57^ Importantly, Meijboom and colleagues^57^ have shown that injection of an AAV9 expressing Cas9 and gRNAs (deleting the repeats) led to reduction of RNA foci and dipeptide repeat proteins in transgenic ALS mouse models harboring 100–1,000 *C9orf72* expanded repeats.

## Challenges of genome editing approaches

Genome editing approaches for motor neuron diseases allow permanent modification of endogenous genomic loci and correction of disease-causing mutations (e.g., *SOD1, FUS* and *TARDBP*, *C9orf72*), increasing the expression of therapeutic disease-modifier genes (e.g., *SMN2*), or reducing the expression of disease-causing gene variants (e.g., *SOD1*). However, one of the major obstacles is the limited editing efficiency, which can be due to the low activity of the DNA repair pathway exploited to edit the target cells (e.g., HDR-based approaches in post-mitotic neurons), poor delivery of the CRISPR/Cas9-based enzymes *in vivo* (see below), or a combination of both factors.

Furthermore, Cas nuclease and base editors can have off-target activity at the DNA and/or RNA level, which, however, can be reduced substantially by use of “high-fidelity” enzymes.^58^ Use of Cas9 nuclease can also cause generation of large genomic rearrangements.^30^ DSB-free BE approaches drastically reduce this genotoxic risk;^59^ however, bystander editing remains a concern, although novel, more precise enzymes have been developed.^58^ PE approaches would avoid bystander editing while allowing all 12 possible base conversion; therefore, PE could potentially be a promising future therapeutic strategy for treating motor neuron diseases.

*In vivo* delivery of CRISPR/Cas9-based systems is also challenging. AAV vectors can be used to deliver CRISPR/Cas9 systems to target cell populations in the CNS, including glia, interneurons, and motor neurons. However, AAV delivery is limited because these vectors cannot accommodate transgene cassettes larger than 4.9 kb. Therefore, in HDR-based approaches, the Cas9 nuclease, gRNA, and donor cannot fit in a single AAV. Furthermore, AAVs cannot accommodate large base editors. Therefore, many groups explored use of smaller Cas9 enzymes (e.g., SaCas9^44^^,^^45^ and SauriCas9^60^) or two AAVs carrying the different components of these CRISPR/Cas9-based systems.^46^^,^^47^ However, optimization of dual AAV transduction and amelioration of the intein systems are still required to reach therapeutically relevant editing efficiency in target neuronal populations. Although delivery of CRISPR-Cas9-based systems is challenging, these genome editing approaches will generate permanent changes in the genome, potentially providing more definitive treatments compared with AAV gene addition strategies.

Furthermore, the immunogenicity of AAV vectors and of CRISPR/Cas9-based bacterial systems is a major concern. Pre-existing immunity against the AAV and Cas9 is possible, and this hampers the use of specific AAV serotypes and Cas9 or Cas9-based enzymes.^61^^,^^62^ Furthermore, in the absence of pre-existing immunity, the AAV and Cas9 can elicit an immune response, which could reduce therapeutic efficacy and prevent re-administration of the drug product, although, in most cases, the hope is to reach a sufficient therapeutic effect with a one-time treatment. Importantly, the AAV can persist for a long time in the body, determining a continuous expression of Cas9 or Cas9-based bacterial enzymes. Therefore, more transient delivery methods are under development, such as lipid- or polymer-based nanoparticle delivery of Cas9-based systems as RNA or ribonucleoproteins.^63^

Finally, although a universal therapy restoring *SMN2* splicing is feasible for SMA, the heterogeneity of genes and mutations causing ALS does not allow development of a unique therapeutic product. Therefore, in most cases, personalized therapeutic strategies need to be developed for treating ALS, requiring expensive development and investments. Currently, the cost of genetic therapy for motor neuron diseases is extremely high (e.g., $2.1 million for Zolgensma genetic therapy for SMA). However, improved manufacturing and optimization of genome editing tools will likely lead to a significant price reduction for these genetic therapies in the next years. Furthermore, a major advantage of genome editing-based strategies is the potential for one-time treatment compared with constant replenishment for the current oligonucleotide-related therapies.

## Conclusions

Overall, the initial translational research studies discussed in our review offer great hope for successful use of innovative genetic therapy approaches to target ALS- and SMA-related mutations. However, as discussed above, important work needs to be accomplished prior to entering clinical trials based on genome editing strategies. It will also be quite important to determine whether these approaches could be combined with pharmacological interventions for SMA and ALS to enhance their therapeutic efficacy. Recent research using animal models and cells from affected individuals has shown significant advances and promising results, which can lead to initiation of clinical trials that could lower the progression of these deadly disorders.
